# Long-Term Results and Current Problems in Laparoscopic Gastrectomy: Single-Center Experience

**DOI:** 10.1089/lap.2020.0180

**Published:** 2020-11-02

**Authors:** Cemil Yüksel, Ogün Erşen, Ümit Mercan, Salim İlksen Başçeken, Batuhan Bakırarar, Sancar Bayar, Ali Ekrem Ünal, Salim Demirci

**Affiliations:** ^1^ General Surgery Department, Surgical Oncology Clinic, Ankara University Medicine Faculty, Ankara, Turkey.; ^2^ Surgical Oncology Clinic, Diyarbakır Research Hospital, Diyarbakir, Turkey.; ^3^ Biostatistic Department, Ankara University Medicine Faculty, Ankara, Turkey.

**Keywords:** gastrectomy, laparoscopy, oncology, survival

## Abstract

***Introduction:*** The study aims to evaluate the long-term results of patients who underwent laparoscopic gastrectomy for gastric cancer in Ankara University Medical Faculty, Surgical Oncology Clinic, within 5 years.

***Materials and Methods:*** We retrospectively reviewed the data of patients who underwent laparoscopic gastrectomy for gastric cancer at the Surgical Oncology Clinic of Ankara University Medical Faculty between January 2014 and September 2019. One hundred forty-six patients were included in the study.

***Results:*** Fifty-one (34.9%) of the patients were female; 95 (65.1%) were male. The mean ± standard deviation and median (minimum–maximum) values of the patients were 60.92 ± 14.13 and 64.00 (22.00–93.00), respectively (Table 1). Eighty-seven (59.6%) cases were located in the antrum, 29 (19.9%) were in the cardia region, and 30 (20.5%) were in the corpus region. Overall, 106 (72.6%) of 146 patients were alive, while 40 (27.4%) were ex. The mean survival was 21.8 months (0–69). Postoperative mortality was seen in 9 patients (6.2%) and our disease-free survival rate was 70.5%. Recurrence occurred in 14 (9.6%) of all patients.

Patient Characteristics

***Conclusion:*** In conclusion, although laparoscopic gastrectomy is a reliable and feasible method for gastric cancer, the standardization of laparoscopic surgery is required in clinics.

**Table 1.** Patient Characteristics

Variables	
Age	
Mean ± SD	60.92 ± 14.13
Median (min.–max.)	64.00 (22.00–93.00)
Albumin	
Mean ± SD	3.90 ± 0.51
Median (min.–max.)	4.00 (2.50–5.01)
Met lymph count	
Mean ± SD	8.41 ± 7.05
Median (min.–max.)	6.00 (1.00–40.00)
Total lymph count	
Mean ± SD	23.09 ± 8.97
Median (min.–max.)	22.00 (4.00–52.00)
Survival	
Mean ± SD	21.83 ± 17.40
Median (min.–max.)	17.50 (0.10–69.00)
Gender, *n* (%)	
Man	95 (65.1)
Woman	51 (34.9)
Morbidity, *n* (%)	
No	117 (80.1)
Yes	29 (19.9)
TM location, *n* (%)	
Antrum	87 (59.6)
Cardia	39 (19.9)
Corpus	30 (20.5)
Operation, *n* (%)	
Subtotal	103 (70.5)
Total	43 (29.5)
Anastomosis, *n* (%)	
Open	6 (4.1)
Intracorporeal	54 (37.0)
Mini-laparotomy	86 (58.9)
Sur comp, *n* (%)	
No	131 (89.7)
Yes	15 (10.3)
T, *n* (%)	
1	32 (21.9)
2	18 (12.3)
3	55 (37.7)
4	41 (28.1)
N, *n* (%)	
Negative	68 (46.6)
Positive	78 (53.4)
Mortality, *n* (%)	
No	106 (72.6)
Yes	40 (27.4)
Recurrence, *n* (%)	
No	132 (90.4)
Yes	14 (9.6)

## Introduction

Gastric cancer is one of the most common malignancies in the world, and^1^ gastrectomy and proper lymph node dissection have been known to improve survival in gastric cancer for many years. The popularity of laparoscopic gastrectomy is increasing. The first laparoscopic-assisted subtotal gastrectomy in history was performed in 1994^2^ and the use of laparoscopy has increased over the years as technology advances. Laparoscopic gastrectomy has become a common cause of less postoperative pain, shorter hospital stays, early return of bowel functions, as well as oncologic outcomes similar to open surgery.^3,4^ Laparoscopic gastrectomy is a difficult surgical procedure and various techniques and different studies are available. In a study by Chen et al., laparoscopic gastrectomy was shown to have advantages over open surgery^5^; Lee et al. reported that there were more anastomosis leakage and higher mortality in laparoscopic surgery.^6^ The safety and efficacy of laparoscopic subtotal gastrectomy (LASG) have been proved in worldwide studies,^7^ whereas the safety and efficacy of laparoscopic total gastrectomy (LATG) have not been proved compared to open surgery. The preferred reason for LATG is that in particular, the reconstruction stage includes some technical limitations in conventional surgery.^8^ The study aims to evaluate the long-term results of patients who underwent laparoscopic gastrectomy for gastric cancer in Surgical Oncology Clinic, Ankara University Medical Faculty within 5 years.

## Materials and Methods

### Selection of patients

We retrospectively reviewed the data of patients who underwent laparoscopic gastrectomy for gastric cancer at the Surgical Oncology Clinic of Ankara University Medical Faculty between January 2014 and September 2019. Patients younger than 18 years of age, stage 4, underwent additional visceral organ resection (liver, pancreas, colon, and small intestine), operated for malignant stromal and neuroendocrine tumors, operated under emergency conditions and previously operated for another malignancy were excluded from the study. One hundred forty-six patients were included in the study.

### Data collection

Diagnostic laparoscopy was performed in 68 patients whose operability could not be clarified with preoperative evaluations. Surgical and pathological reports, preoperative hematological and biochemical parameters, tumor markers, demographic characteristics, postoperative complications, postoperative mortality, and total disease-free survival were evaluated. Radiological examinations (chest radiography, computed tomography, ultrasonography, endoultrasonography, magnetic resonance, and positron emission tomography) were examined by scanning retrospectively the electronic files. For staging, the eighth edition of the TNM classification for International Cancer Control^9^ and the Japanese Gastric Cancer Association guidelines^10^ were used. One-seven lymph node stations in D1 dissection and lymph nodes in D1 + 8a, 9, 10, 11p, 11d, and 12a stations in D2 dissection were included. Complications were defined according to the Clavien–Dindo classification. Postoperative complications were accepted as complications that occurred within 90 days and postoperative mortality was accepted as patients who died within 30 days or during hospitalization.

### Surgical procedure

**FIG. 1.**
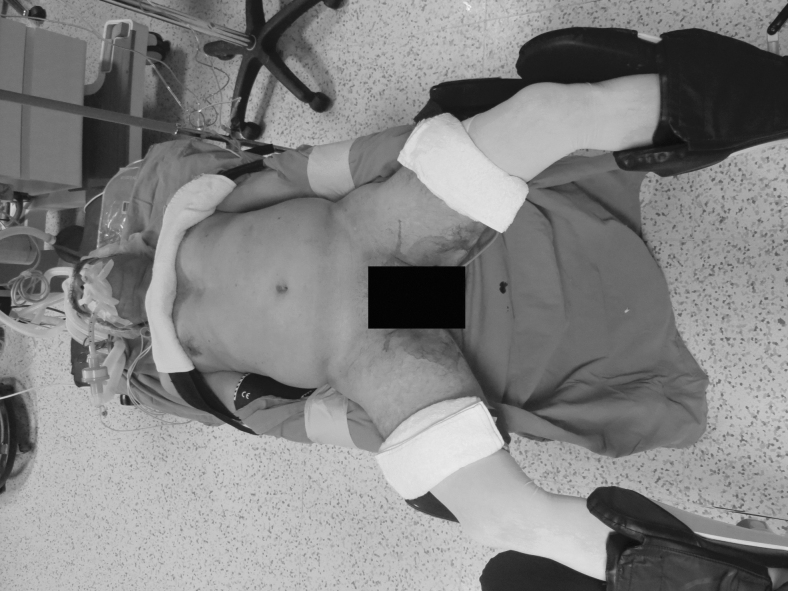
Patient position.

**FIG. 2.**
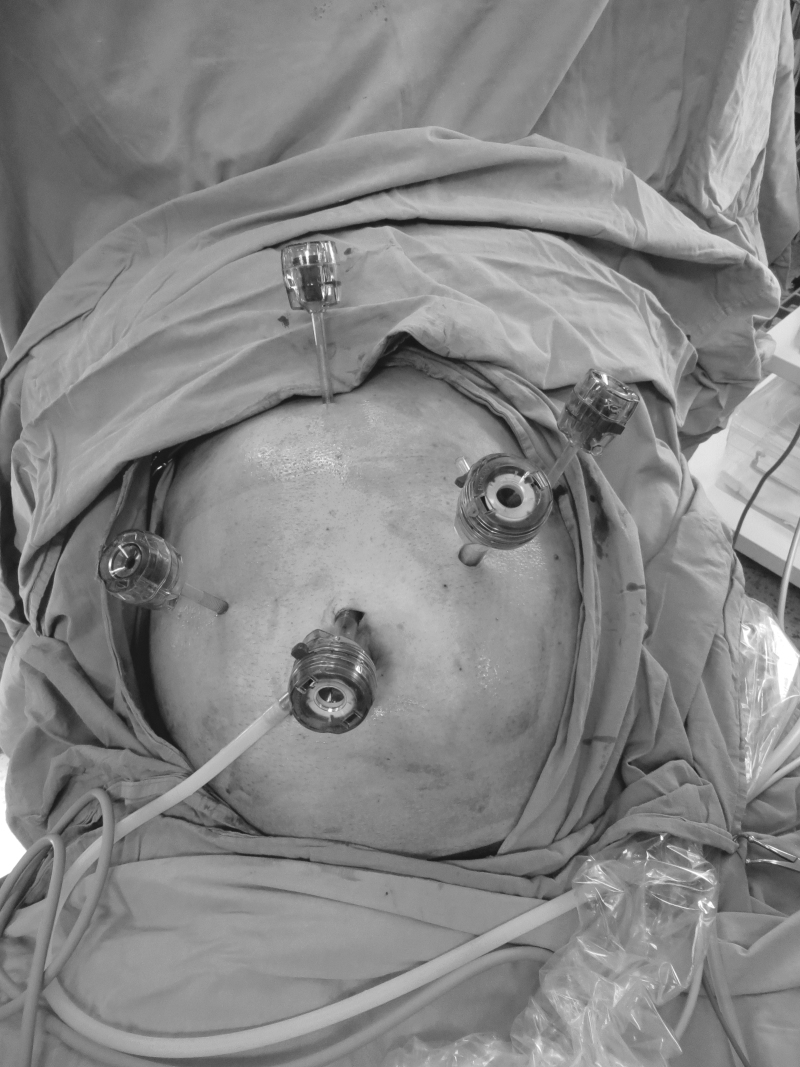
Port entry places.

**FIG. 3.**
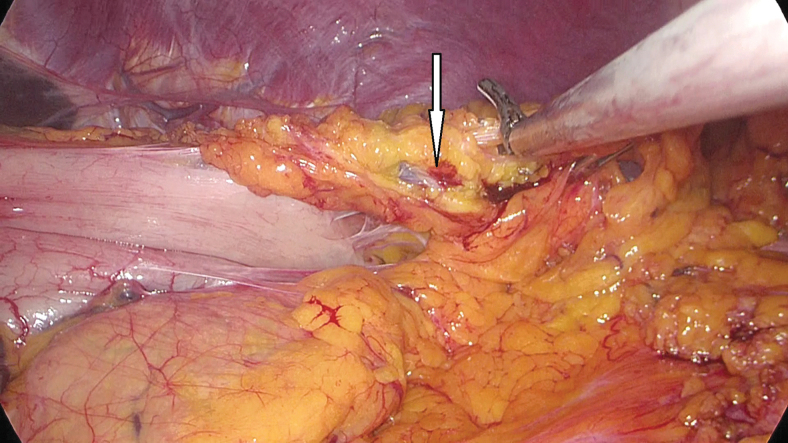
Left gastroepiploic artery (arrow).

**FIG. 4.**
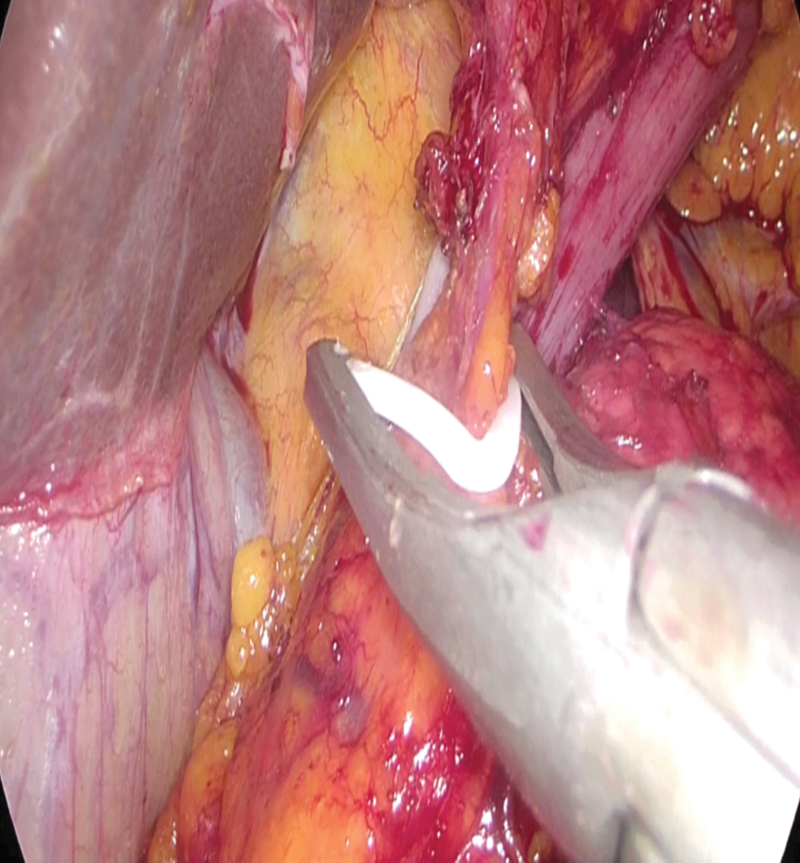
Clipping of right gastroepiploic artery and vein.

**FIG. 5.**
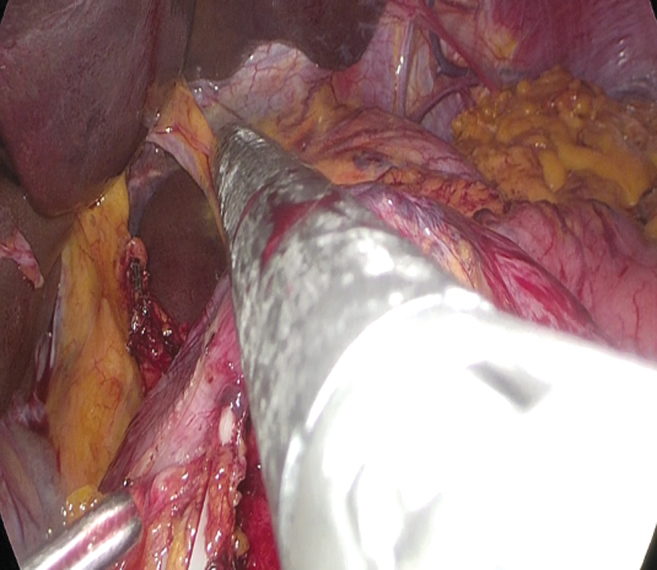
Cutting the duodenum.

**FIG. 6.**
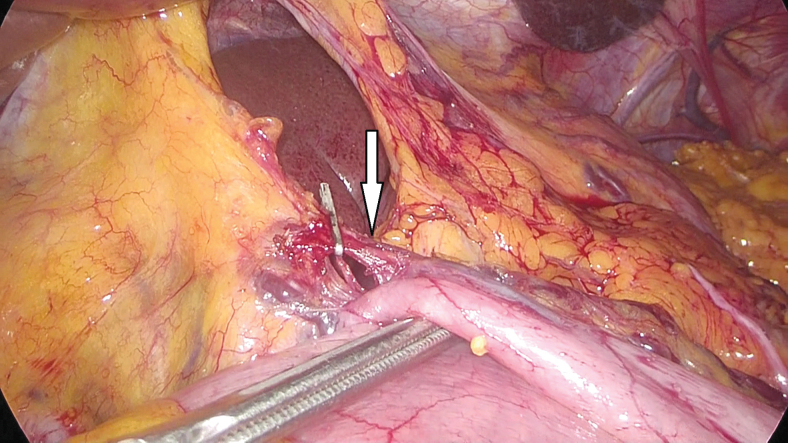
Right gastric artery ligation (arrow).

**FIG. 7.**
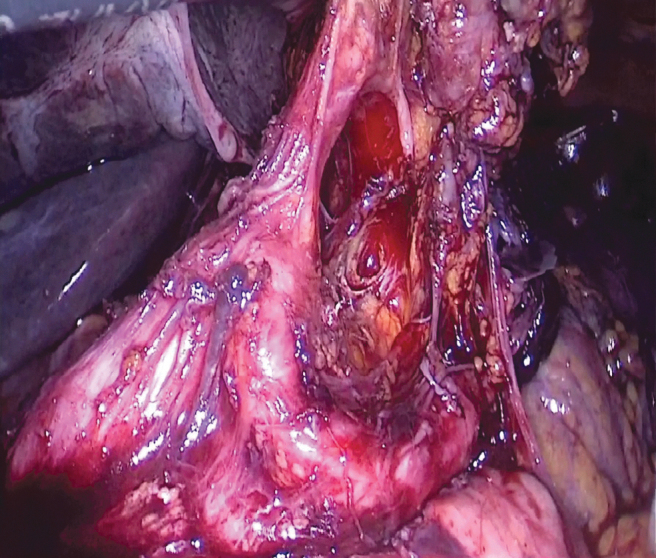
Dissection of the celiac trunk.

**FIG. 8.**
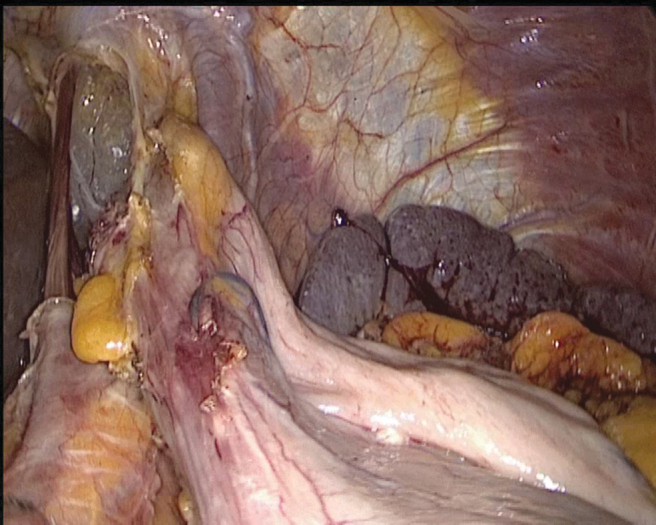
Dissection of the paracardiac region.

The same surgical team performed all cases. Preoperative informed consent was obtained from the patients for the operation. Intestinal cleaning was performed using laxatives and enemas 1 day before the operation and the patient was operated after 8-hour fasting. Preoperative single-dose prophylactic antibiotic (cefazolin 1 g) was administered. Intraoperative normothermia was provided by anesthesiologists and all surgical procedures were performed according to routine asepsis and antisepsis rules. All patients were placed in the opposite Trendelenburg and French positions by closing both arms (Fig. 1). The position was changed according to the stages of the surgery, but the position was mainly on the right side. We entered via a 12 mm camera trocar (PT00015248; Medtronic, Dublin, Ireland) directly over the umbilicus. Pneumoperitoneum was maintained with a mean of 12–15 mmHg. After the pneumoperitoneum was created, a 5 mm trocar for the left hand of the surgeon, 2 cm above and 5 cm left lateral of the camera port, 12 mm trocar for the right hand, 2 cm above the camera port, and 5 cm right lateral were entered into the abdominal cavity. For the assistant, a 5 mm trocar was entered from the lateral axis of the right 12 mm trocar through the intersection of the middle axillary line, and finally, an automatic liver retractor (G03123; Cook-Medical, Bloomington) was placed in the subxiphoid region (Fig. 2). A 30° camera and a laparoscopy system capable of displaying two- or three-dimensional images were used in all cases (OTV-S300; Olympus, Norfolk). After positioning the patient and entering the trocars, exploration was performed and the presence of distant metastasis or peritoneal spread was examined carefully. Then, the gastrocolic ligament was opened with ultrasonic scissors (laparoscopic coagulating shears; Ethicon Endo-Surgery, Cincinnati, OH) and proceeded until the spleen was seen on the left, and the left gastroepiploic artery was ligated at the root of the vein. Number 4 lymph nodes were included in the specimen (Fig. 3). If total gastrectomy is performed, it is reached to the left crus. On the right side, we proceeded till the duodenum and gall bladder. Above the duodenum, fat was released from the tissues, and the right gastroepiploic artery and vein were ligated and cut with hem-o-loc clips (Weck Closure System; Teleflex Medical, Research Triangle Park, NC) and the infrapyloric lymph nodes (number 6) were included in the specimen (Fig. 4). Then the duodenum was transected with a 45 mm endoscopic stapler instrument (030459; Medtronic) 2 cm below the pylor. Then (Fig. 5), the right gastric artery was ligated and cut on the hepatic artery (Fig. 6). The hepatoduodenal ligament was first removed from the posterolateral peritoneum on the portal vein (12p) and the lymph glands were collected, then the pancreas capsule was included and the hepatic artery (number 8a) was cleaned and ligated with the clips of the left gastric artery and vein (number 7). The lymph nodes on the proximal splenic artery were dissected (number 11p) starting from the celiac trunk (Fig. 7) and lymph nodes on the distal splenic artery were included in the specimen (number 11d). From here, we reached the spleen and collected lymph nodes on the vascular structures (number 10). The next step was to include lymph nodes around the small curvature (number 3) and dissection of the lymph nodes in the right paracardiac region was completed (Fig. 8).

**FIG. 9.**
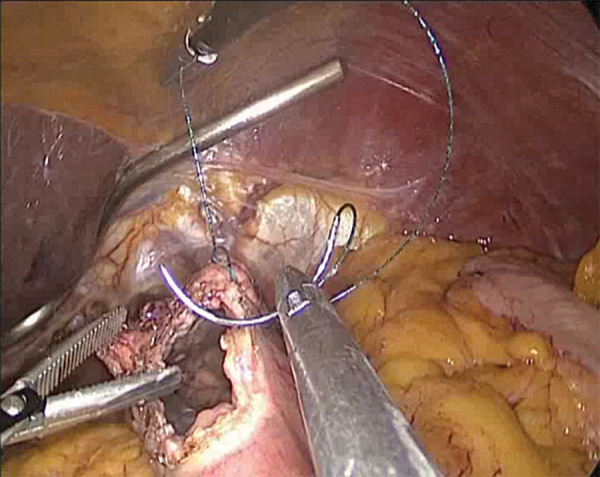
Suturing the stomach wall with a barbed stitch laparoscopically.

All patients underwent Roux-en-Y gastrojejunostomy or esophagojejunostomy for reconstruction. From the Treitz, the jejunum was transected from the 20 cm and the distal part was anastomosed to the stomach or esophagus, while the Y leg was anastomosed to the 40 cm. Some of these anastomoses were performed intracorporeally and some were performed by mini-laparotomy. Mini-laparotomy was performed with a 5 cm incision in the midline just below the xiphoid. The incision template (Alexis Wound retractor; Applied Medical, Rancho Santa Margarita, CA) was used to minimize skin contact with the incision site. Intracorporeal esophagojejunostomy was performed with a linear stapler and overlap anastomosis. After that, the stapler inlet openings were sutured in double layers (Fig. 9).

The circular stapler was used when mini-laparotomy was performed in the anastomosis. Intracorporeal gastrojejunostomy anastomosis was performed side-by-side with a linear stapler or double-sided, end-to-side by hand. In patients who underwent mini-laparotomy, anastomoses were performed by stapler or by hand. Two drains were placed in the patient who underwent total gastrectomy and one drain was placed in the patient who underwent subtotal gastrectomy.

### Statistical analysis

SPSS for Windows 11.5 program was used for data analysis. The mean ± standard deviation and median (minimum–maximum) were used for the quantitative variables and the number of patients (percentage) for the qualitative variables. Logistic regression analysis was used to evaluate the risk factors affecting the dependent qualitative variable. Survival analyses on qualitative and quantitative variables were performed using the Kaplan–Meier method, and statistical significance between the groups was established using the log-rank test. Multivariate analyses were performed using the Cox proportional hazard model to examine the factors affecting survival. In the univariate Kaplan–Meier analysis, significant variables (*P* < .05) were included in the Cox proportional hazard model. Statistical significance accepted as 0.05.

## Results

One hundred forty-six patients underwent laparoscopic gastrectomy for gastric cancer. Fifty-one (34.9%) of the patients were female; 95 (65.1%) were male. The mean ± standard deviation and median (minimum–maximum) values of the patients were 60.92 ± 14.13 and 64.00 (22.00–93.00), respectively (Table 1). Eighty-seven (59.6%) cases were located in the antrum, 29 (19.9%) were in the cardia region and 30 (20.5%) were in the corpus region. There was no statistical significance between tumor localization and survival. Overall, 106 (72.6%) of 146 patients were alive, while 40 (27.4%) were ex. The mean survival was 21.8 months (0–69). Postoperative mortality was seen in 9 patients (6.2%) and our disease-free survival rate was 70.5%. Recurrence occurred in 14 (9.6%) of all patients. Oral enteral nutrition was started on a mean 3.32 days.^1–15^ The length of hospital stay was 8.8 days, and if postoperative mortality was excluded, it ranged from 4 to 50 days.

Univariate logistic regression analyses are shown in Table 2. The ratio of the number of metastatic lymph nodes and the number of metastatic lymph nodes to the total number of lymph nodes was found to be significantly associated with poor survival (*P* < .05). Also, when the number of metastatic lymph nodes was increased by 1 unit, the negative effect on survival was 1.084, whereas the ratio of the metastatic lymph node to total lymph node by 1 unit had a 7.842-fold negative effect on survival.

**Table 2.** Univariate Logistic Regression for Mortality

Variables (references)	β	SE	P	OR	95% Confidence interval	
					Lower limit	Upper limit
Met lymph count	0.081	0.038	**.034**	1.084	1.006	1.168
Total lymph count	−0.011	0.021	.596	0.989	0.949	1.031
Met/total	2.059	0.915	**.024**	7.842	1.306	47.093
CA19-9	−0.347	0.287	.227	0.707	0.402	1.241
CEA met lymph	0.002	0.004	.571	1.002	0.994	1.011

Bold indicates statistically significant.

CEA, carcinoembryonic antigen; OR, odds ratio; SE, standard error.

Table 3 shows the univariate Kaplan–Meier analysis results and the 1-, 3-, and 5-year survival probabilities of the variables affecting survival. The mean ± standard error, median ± SD values, and *P* values that test their statistical significance for these periods are indicated.

**Table 3.** Kaplan–Meier Analysis Results

Variables	Survival					P
	1 Year (%)	3 Years (%)	5 Years (%)	Life time		
				Mean ± SD	Median ± SD	
General	81.8	64.3	61.8	48.57 ± 2.68	—	—
Morbidity						
No	86.9	68.2	64.7	51.30 ± 2.91	—	**.003**
Yes	62.1	48.9	48.9	36.04 ± 6.08	29.00 ± —	
TM location						
Antrum	80.5	58.3	58.3	46.25 ± 3.52	—	.498
Cardia	81.9	66.4	—	43.47 ± 4.60	—	
Corpus	85.5	85.5	—	45.63 ± 4.11	—	
Anastomosis						
Open	50.0	—	—	16.77 ± 5.50	11.00 ± 6.89	**.038**
Intracorporeal	81.2	64.5	—	32.83 ± 2.62	—	
Mini-laparotomy	84.5	68.2	65.3	50.65 ± 3.21	—	
Conversion						
No	83.9	67.0	64.3	50.08 ± 2.70	—	**.002**
Yes	42.9	21.4	—	21.16 ± 8.78	11.00 ± 7.20	
Anal leakage						
No	81.6	65.9	63.2	49.15 ± 2.74	—	.479
Yes	85.7	42.9	—	36.66 ± 8.99	29.00 ± 5.23	
Surgical complication						
No	82.0	66.3	63.4	49.51 ± 2.82	—	.294
Yes	80.0	49.4	49.4	39.77 ± 7.93	29.00 ± —	
Surgical margin						
Negative	84.8	66.6	64.0	50.16 ± 2.68	—	**<.001**
Positive	16.7	—	—	6.87 ± 3.00	3.00 ± 3.61	
Met lymph						
No	89.4	86.7	86.7	60.71 ± 2.75	—	**<.001**
Yes	76.1	45.5	—	34.45 ± 3.09	29.00 ± 7.40	
T						
1	90.6	85.3	85.3	60.40 ± 4.01	—	**<.001**
2	88.9	88.9	88.9	53.34 ± 4.44	—	
3	92.2	77.2	71.3	54.51 ± 3.75	—	
4	59.7	23.9	—	22.67 ± 3.26	15.00 ± 5.78	
Early-local						
Locally advanced	79.4	58.8	56.0	44.96 ± 3.06	—	**.043**
Early gastric	90.9	85.6	85.6	60.58 ± 3.94	—	
Recurrence						
No	83.2	73.4	70.6	52.77 ± 2.64	—	**<.001**
Yes	70.1	0.00	—	18.53 ± 3.12	15.00 ± 1.18	
Lymphovascular invasion						
No	94.4	91.9	91.9	64.15 ± 2.34	—	**<.001**
Yes	72.9	45.6	42.0	36.78 ± 3.45	29.00 ± 7.55	
Perineural invasion						
No	95.0	86.5	86.5	61.54 ± 2.50	—	**<.001**
Yes	67.5	39.7	35.3	33.06 ± 3.83	28.00 ± 6.68	
Albumin						
Low	56.4	20.2	20.2	23.58 ± 4.69	15.00 ± 2.58	**<.001**
Normal	89.8	79.9	76.3	57.19 ± 2.63	—	
RDW						
Normal	93.3	85.9	85.9	61.26 ± 2.31	—	**<.001**
High	58.2	26.8	22.3	25.50 ± 4.00	16.00 ± 3.50	

Bold indicates statistically significant.

RDW, red cell distribution width; SD, standard deviation; TM, tumor.

Normal red cell distribution width (RDW) value of the patients was accepted as 11.5%–14.5%, and there were 48 patients (32.8%) with 14.5% or more. There was statistical significance between RDW height and prognosis (*P* < .001). Albumin values of 3.5 g/dL and above were accepted normal and below 3.5 g/dL were accepted hypoalbuminemia. The number of patients with hypoalbuminemia was 33 (22.6%). Survival was poor in patients with hypoalbuminemia (*P* < .001). Among the tumor markers in the preoperative period, CA19-9 was found high in 22 (15.06%) patients and carcinoembryonic antigen (CEA) in 23 (15.75%) patients. While CA19-9 was statistically significant (*P* = .001), CEA had no statistically significant (*P* = .143) relationship with prognosis. Tumor markers and metastatic lymph node ratio effect on survival was not statistically significant (*P* > .05).

LASG was performed in 103 patients (70.5%), LATG and D2 dissections in 43 patients (29.5%). In patients with LATG, anastomosis leakage was higher than those with LASG, and a statistically significant difference was found between these two groups (*P* = .013). In patients who underwent LATG, hospitalization stay and oral enteral initiation were longer, and a statistically significant difference was found between the two groups (*P* = .003 and *P* = .001). There was no statistical significant in terms of overall survival, disease-free survival, and deficit conversion (*P* > .05).

Anastomoses were performed by intracorporeal or mini-laparotomy. After mini-laparotomy was performed in 86 (58.9%) of the patients, 54 (37%) were performed intracorporeal anastomosis. The anastomosis of 6 patients who were converted from laparoscopy was also performed after the open conversion. Roux-en-Y anastomosis was performed in all patients. Longer survival was observed in patients who underwent anastomosis after mini-laparotomy, and it was found statistically significant (*P* = .038).

Six of the patients (6.1%) had an open conversion. Open conversion causes were peri-organ invasion (colon and pancreas) in 2, bulky lymph nodes in 1, left replaceable hepatic artery injury in 1, and esophagus mobilization in 2 patients. These patients had worse prognosis (*P* = .02).

Postoperative complications occurred in 18 patients. While 2 of the 3 patients with duodenal stump leak improved with medical treatment, the other patient was operated again. Pleural effusion occurred in 1 patient and catheter was inserted into the pleural cavity and drainage was achieved. Colon resection was performed in 1 patient who developed transverse colon ischemia. Anastomotic stenosis and proximal surgical margin positivity developed in 1 patient, and both had complementary total gastrectomy. In 2 of the patients, the intraabdominal abscess was developed and the catheter was inserted into the abdominal cavity by interventional radiology. When postoperative bleeding occurred in 2 patients, 1 patient was operated again while the other was followed. Anastomosis leakage occurred in 7 patients. One of the leaks was enteroenterostomy, 5 were esophagojejunostomy, and 1 was gastroenterostomy anastomosis. While most patients with anastomosis leakage were undergoing revision surgery, 2 improved with medical treatment, 1 from esophagojejunostomy and the other from gastroenterostomy. There was no statistically significant relationship between anastomosis leak and prognosis (*P* > .05). There was no statistically significant difference found between prognosis and all surgical complications (*P* > .05). Seven patients were reoperated due to complications, and their prognosis was worse (*P* = .05).

We classified the patients according to the Clavien–Dindo classification system in terms of postoperative morbidity. While there were 100 patients in group 1, there were 22 in group 2, 8 in group 3a, 7 in group 3b, 1 in group 4, and 8 in group 5. Likewise, we divided the patients into two groups as medium (Clavien–Dindo 1–2) and severe (Clavien–Dindo 3a–3b–4). While 122 patients were in the middle group, 16 patients were in the heavy group. As a result of the statistical analysis conducted, the survival significantly decreases as you move from the middle group to the heavy group or move from group 1 to other groups (morbidity increases) (*P* = .003).

According to the pathology reports, 84 (57.5%) of the patients had lymphovascular invasion (LVI) and 64 (43.8%) had perineural invasion (PNI). The presence of LVI and PNI showed poor survival (*P* < .001). The surgical margin was positive in 6 patients, and the proximal and distal surgical margins were positive in 3 patients. Only one of these patients was reoperated; others were directed to medical oncology after discussion at the council due to age, additional comorbidities, and postoperative complications. A statistically significant difference was found between the surgical margin positivity and prognosis (*P* < .001). The average number of metastatic lymph nodes was 8.41 (0–40) and the average number of total lymph nodes was 23.08 (4–52). Mortality was higher in patients with metastatic lymph nodes (*P* < .001). When classified according to T stage, 32 patients were T_1_ (21.9%), 18 patients were T_2_ (12.3%), 55 patients were T_3_ (37.7%), and 41 patients were T_4_ (28.1%). It was observed that survival decreased as the T stage increased (*P* < .001). The proportions of patients with T_2_ and T_3_ stages were very close. N was positive in 78 (53.4%) of patients and N was negative in 68 (46.6%). Mortality was found to be high in patients with N-positive results and was statistically significant (*P* < .05). When T_2_N_0_ and T_2_N+ patients were compared, no statistical difference was found between the two groups (*P* > .05). When T_3_N_0_ and T_3_N+ patients were compared, no statistical difference was found between the two groups (*P* > .05).

We divided our patients into two groups as early and local advanced gastric cancer. We defined early gastric cancer as T_1_N_x_M_x_ and all other patients as locally advanced gastric cancer. Thirty-three (22.6%) of the patients were with early gastric cancer, and 113 (77.4%) were with locally advanced gastric cancer. There was a statistically significant difference in survival between the two groups and was lower in locally advanced gastric cancers (*P* = .026). There was no statistically significant difference between the two groups in terms of anastomosis leakage, postoperative mortality, anastomosis technique, and oral feeding, but postoperative mortality was also *P* = .055; 88% of cases were with locally advanced gastric cancer and 12% of cases with early gastric cancer. Although not statistically significant, it was found significantly higher in locally advanced cancers. In general, survival was found to be higher in early gastric cancers and it was statistically significant (*P* = .043). Sixteen (11%) of the patients received neoadjuvant therapy. As neoadjuvant therapy, FOLFOX (Folinic acid+Fluorouracil+Oxaliplatin), FLOT (Folinic acid+Fluorouracil+Oxaliplatin+Taxane), DCF (Docetaxel+Fluorouracil+Cisplatin), Carboplatin, and Paclitaxel treatment regimens were performed. It was statistically found that survival was worse in patients receiving neoadjuvant therapy (*P* = .046). At least one blood product (erythrocyte, fresh frozen plasma, and platelet) was used in 33 (22.6%) of the patients and it was found that it had a negative effect on total survival (*P* < .001).

In these 5 years, we divided the cases into two groups as the first 3 and the last 2 years. Twenty (29%) of 69 (46.2%) patients operated in the first 3 years and 20 (26%) of 77 (53.8%) patients operated in the last 2 years were exes. There was no statistically significant difference between the two groups in terms of overall mortality, postoperative mortality, and anastomosis leakage (*P* > .05), while survival was statistically better in the first 3 years (*P* < .05).

In the multivariate analysis according to the Cox regression analysis, open conversion, LVI, PNI, and hypoalbuminemia were found statistically significant in terms of prognosis (*P* < .001). The morbidity, anastomosis, open conversion, surgical margin, met lymph, T, early local, recurrence, LVI, PNI, albumin, and RDW variables that were significant as a result of the Kaplan–Meier analysis in Table 3 were included in the Cox regression analysis.

Considering all the descriptive variables in the study together, the results of the model were obtained when Cox regression analysis was performed with the backward selection method to evaluate which factors affect survival. As can be seen in Table 4, open conversion, LVI, RDW, and Albumin variables remained in the model (*P* < .005). Patients with open conversion are 3.27 times more at risk than those without open conversion; those with LVI are 5.28 times more at risk than those without, those with RDW elevated are 3.55 times more at risk than those without, and patients with hypoalbuminemia are 4 times more at risk than those without.

**Table 4.** Cox Regression Analysis

Variables	Survival			
	Hazard ratio	95% Confidential interval		P
		Lower limit	Upper limit	
Conversion				
No	1 (reference)	—	—	—
Yes	3.270	1.257	8.505	.015
Lymphovascular invasion				
No	1 (reference)			—
Yes	5.280	1.803	15.457	.002
RDW				
Normal	1 (reference)	—	—	—
Elevated	3.511	1.628	7.571	.001
Albumin				
Normal	1 (reference)			—
Low	4.146	2.096	8.202	<.001

RDW, red cell distribution width.

## Discussion

Gastrectomy and lymphadenectomy must be done for curative resection in gastric cancer.^11^ Many studies have been published recently stating that laparoscopic surgery is superior to open surgery.^12,13^ Laparoscopic gastrectomy is becoming more and more common and the reasons are short-term similar oncological results, faster return to normal postoperatively, early gained bowel functions, less pain, and less wound infection development.^14,15^ The fact that reconstruction requires experience and difficulty of lymphadenectomy are the factors that make laparoscopy difficult. As the experience increases, reconstruction and lymphadenectomy will be performed safer. Depending on these, use of laparoscopy in gastric cancer surgery will become common.

In the literature, postoperative morbidity is around 4%–27%^16^ in cases with open gastrectomy, and on average, it is around 8%–24% in cases with laparoscopic gastrectomy.^17–20^ In the literature, morbidity of open and laparoscopic gastrectomy was compared and similar rates were found.^21^ The mortality rate after open surgery in the literature is 0%–6.6%.^22,23^ In our study, the postoperative morbidity rate was 19.9% and the mortality rate was 6.2%. Kitano et al. showed that the rates of morbidity and mortality in early gastric cancers were 14.8%–0%, respectively,^24^ Fujiwara et al. reported 22.3%–0%, respectively.^25^ In our series, the morbidity and mortality rate in early gastric cancer was found 18.18% to 3.03%, respectively. In our study, the postoperative complication rate was found as 10.3%. Morbidity and mortality do not increase in experienced centers for laparoscopic surgery, moreover, morbidity and mortality are lower in these centers. All these show us that laparoscopic gastrectomy does not increase morbidity and mortality.

Anastomosis leaks cause major morbidity and mortality after gastrectomy. In the literature, the rate of anastomosis leakage after laparoscopic surgery is around 1.1%–2.7%.^26^ In our study, the rate of anastomosis leakage was 4.8%, as close to the literature. The reason why it was more than reported in the literature is that the locally advanced gastric cancer was higher. In early gastric cancer patients, anastomosis leakage was 0%, in the same with the literature. In our study, anastomosis leaks were slightly higher after LATG and that was not statistically significant (*P* > .05). The reason for this may be that conventional esophagojejunostomy was a difficult anastomosis performed in a narrow area. There was no statistically significant relationship between anastomosis leak and prognosis (*P* > .05). This indicates that anastomosis leaks can be detected early and can be overcome with an appropriate treatment approach, and as a consequence of these, the prognosis remains unaffected. Lee et al. reported less bleeding in the anastomosis area in LATG.^27^ No bleeding from the anastomosis area was observed in any of the patients included in our study. This shows that better exploration will be achieved by laparoscopy in a narrow anastomosis region and more rigorous hemostasis can be achieved in laparoscopy. In some studies in the literature, it has been reported that pancreatic fistula rates are high, especially after conventional surgery.^28^ There were no pancreatic fistula cases in our study. The reason for the less pancreatic fistula in laparoscopic surgery may be to perform lymph node dissection at a larger magnification than open surgery.

Studies have shown that the length of hospital stay after laparoscopic surgery is shorter in laparoscopic surgery and postoperative complications are similar to those of open surgery.^29,30^ In our study, the hospital stay is on average 8.8 days and there is no increase in complications. Lee et al. compared laparoscopic distal/total gastrectomy and showed that the duration of hospitalization was slightly longer in total gastrectomy.^31^

In our study, it was observed that the patients who had LATG had a longer hospital stay. We believe that LATG can be done safely in experienced centers by following oncological principles without increasing complication rates although it takes a little longer.

In the literature, the 5-year survival rate in patients undergoing laparoscopic gastrectomy is around 95%.^21,32,33^ In our study, this rate was 72.6%. The reason why this rate was less than the literature, early-stage gastric cancers were included in the other study, and in our study, advanced stage gastric cancers were in the majority and patients who were performed at the end of the learning curve had a short follow-up period. In a study of Bo et al.,^34^ the 5-year survival rate in advanced gastric cancers was 49%, while in our study, survival was 68.1% in local advanced cancers and 87.87% in early gastric cancers. This shows that the oncological results of laparoscopic surgery are successful.

In 18 patients with stage T_2_, survival was 88.9%, and survival in 55 patients with T_3_ was 81.8%. These results show us that laparoscopic surgery can be performed safely and effectively in T_2_–T_3_ tumors. As the T stage increases, metastatic lymph nodes increase and survival decreases. Survival rates were T_3_N_0_ 88.9% and T3N+78.4%; T_2_N_0_ is 86.7% and T_2_N + 100%. Today, although there are doubts about the laparoscopic surgical approach in locally advanced gastric cancer, these results show us that laparoscopy can be performed safely in the locally advanced stages as in the early stage, and it is necessary to minimize the doubts with future studies. Lee et al. have reported that laparoscopic surgery was difficult in T_3_ tumors, tumor seeding and port-site recurrence could develop.^35^ We disagree with this. We think that the use of a protective bag when removing the specimen, minimal touch to the tumor, reducing carbon dioxide leakages from the trocars, removing trocars after emptying all the intra-abdominal gas, and closing the fascia at the port sites will prevent tumor seeding and port-site recurrence. Port recurrence has not been observed in our cases up to now.

Recurrence in gastric cancers occurs especially in the liver and peritoneal region. The standard treatment of relapse is not clearly defined. In some studies, the contribution of complementary gastrectomy to survival has been shown.^36,37^ In our study, the recurrence rate was 9.6%. The recurrence rate in gastric cancers with gastrectomy and D2 dissection is between 1.4% and 6.4% in the literature.^38–40^ In our study, the recurrence rate for early gastric cancers was 0%. In locally advanced gastric cancer, it was 12.38%. These results show that laparoscopic surgery does not increase recurrence in gastric cancer and can be safely performed as long as oncological rules are followed.

It is a known fact that the curative results of laparoscopic surgery in early gastric cancers are similar to open surgery. The real question is laparoscopic surgery effectiveness in advanced gastric cancers. In our study, we compared early gastric cancer and advanced gastric cancers in some factors such as postoperative complications, hospitalization, curative resection, and enteral onset, there was no statistical significant difference (*P* > .05). This suggests that laparoscopic surgery will be performed safely in advanced-stage patients. The main suggestion was the idea that lymph node dissection cannot be performed laparoscopically,^2^ but nowadays, lymph node dissection can now be performed at the same rate as open surgery with a minimally invasive approach. Thus, laparoscopic surgery does not have the disadvantage to achieve curative results.

In a study, the number of lymph nodes dissected was stated to be 30 ± 10.^41^ In a similar study, it was stated as 29 ± 11.^42^ In our study, an average of 23.08 ± 8.9 (4–52) lymph nodes were dissected. Lymph node dissection (D2) has been proved to provide a survival improvement in gastriccancer.^43^ The important point here is to dissect a sufficient number of lymph nodes. This allows us to predict optimal staging and patient survival.^44^ In our study, adequate lymph node dissection was performed laparoscopically.

Enteral onset time in the literature is postoperative 3–8 days.^45^ In our study, the average onset of enteral feeding is 3.32 days. We think that there is no difference in laparoscopic or open surgery in enteral onset. In previous studies, it was stated that the duration of hospitalization after laparoscopic gastrectomy was shorter with an average of 7–19 days compared to the open surgery.^46,47^ In our study, the mean duration of hospitalization was 8.87 days, while it was 10.5 days in total gastrectomies and 8.1 days in subtotal gastrectomies, and a statistically significant difference was found between the two groups (*P* = .003). The average conversion rate is 6.1% in our study. While early gastric cancer cases were 3.03%, it was 5.3% in advanced cases. The open conversion rates in the literature range from 2.3% to 25%.^48,49^ As the experience in laparoscopic surgery increases and technology improves, conversion rates decrease significantly.

Studies are indicating that the operation time is between 144 and 348 minutes.^50^ Our average operation time was 142.69 (80–300) minutes. When laparoscopic surgery was just started, the time was expected to be longer. However, as the number of cases increases and standardization is achieved, the duration decreases. In the literature, it has been shown that the time is shorter after the learning curve ends.^51^ In total gastrectomy cases, the duration of surgery was longer than the subtotal ones. We believe that longer operations do not increase complications and minimally invasive surgery should not be abandoned. In our study, high RDW value (14.5% and higher were accepted) was bad prognostic as one of the factors affecting survival or as an indicator (*P* < .001). It is already known that RDW increases in inflammation and affects prognosis. Likewise, low albumin value and CA19-9 elevation were found to be poor prognostic factors (*P* < .001 and *P* = 0.001). Nutritional markers are important for prognosis and postoperative complications, and albumin is used as a nutritional marker in the clinical practice. This shows that we should provide preoperative nutritional support to patients with hypoalbuminemia. In this way, we can reduce the negative effects on prognosis and postoperative morbidity. In our study, the duration from the diagnosis of cancer to the operation was 23.26 days (1–330) and the length of time was extended due to the additional neoadjuvant treatments. In patients without neoadjuvant therapy, the average time from diagnosis to operation was 10.73 days. We think that this time is sufficient. Preoperative blood product was used in 33 patients. These cases were found to have worse survival (*P* < .001). We think that this situation is caused by the immunosuppressive effect of the blood product.

Whether the anastomosis form is intracorporeal or extracorporeal is a matter of preference and does not affect oncological results. In our clinic, an increasing number of intracorporeal anastomoses are performed, and we think that intracorporeal anastomosis is safer. The difficulties of extracorporeal anastomosis are enlargement of the incision due to insufficient mini-laparotomy, intestinal injuries due to difficulty in exploration, postoperative pain especially in obese patients due to the tension applied to the retractor, and late bowel movement. Clear vision can be provided and intestinal injuries are less during laparoscopic anastomosis. The factor that forces us during anastomosis is the surgical margin status, especially in nonpalpable tumors. For this reason, we performed preoperative endoscopy in most cases. After resection, the specimen was examined and the surgical margin was evaluated. In undergoing total gastrectomy patients, we sent the surgical margin to frozen section. Some methods have been developed in the literature to find tumor placement, and preoperative clipping, preoperative ultrasonography,^52^ or preoperative endoscopic staining^53^ are performed. We do not use these methods in our clinic. In the intracorporeal anastomosis technique, we mostly use staplers. We rarely close it manually. In anastomosis made with staplers, we manually close the open space. We do not prefer to use the stapler tool when closing this gap, because we think that otherwise there will be more anastomosis stenosis.

A Korean study stated that the learning curve was 50 cases.^54^ In our clinic, we believe that the learning curve has fewer cases. We think that surgeons who are performing standard laparoscopic surgery (cholecystectomy, hernia repair, and colectomy) can reduce this curve to 20 cases. To achieve this, the surgical procedure should be performed within standardization and accompanied by a senior surgeon. When we differentiate our patients as those operated for the first 3 and last 2 years, the first 3 years' survival was better (*P* < .05). We associated this with the fact that the first 3 years' cases have a longer follow-up period, while in the last 2 years, more patients were operated and the stage was more advanced.

The limitations of our study are the following: it is not randomized, and it is a retrospective and single-center study. Besides, some of the patients have not completed the 5-year follow-up period yet.

In conclusion, although laparoscopic gastrectomy is a reliable and feasible method for gastric cancer, the standardization of laparoscopic surgery is required in clinics. After gaining sufficient experience, curative resection results are achieved with the same or even better oncological results as open surgery. Therefore, laparoscopic gastrectomy is the gold standard in gastric cancer surgery.
